# Organocatalytic Name Reactions Enabled by NHCs

**DOI:** 10.3390/ma13163574

**Published:** 2020-08-13

**Authors:** Krzysztof Dzieszkowski, Izabela Barańska, Karina Mroczyńska, Michał Słotwiński, Zbigniew Rafiński

**Affiliations:** Faculty of Chemistry, Nicolaus Copernicus University in Toruń, 7 Gagarin Street, 87-100 Toruń, Poland; dzieszko@doktorant.umk.pl (K.D.); izabelabaranska@doktorant.umk.pl (I.B.); karina.mroczynska@doktorant.umk.pl (K.M.); michal.slotwinski@op.pl (M.S.)

**Keywords:** name reactions, *N*-heterocyclic carbenes, organocatalysis

## Abstract

Giving reactions the names of their discoverers is an extraordinary tradition of organic chemistry. Nowadays, this phenomenon is much rarer, although already named historical reactions are still often developed. This is also true in the case of a broad branch of *N*-heterocyclic carbenes catalysis. NHCs allow many unique synthetic paths, including commonly known name reactions. This article aims to gather this extensive knowledge and compare historical reactions with current developed processes. Furthermore, this review is a great opportunity to highlight some of the unique applications of these procedures in the total synthesis of biologically active compounds. Hence, this concise article may also be a source of knowledge for scientists just starting their adventure with *N*-heterocyclic carbene chemistry.

## 1. Introduction

Organocatalytic strategies enabled by *N*-heterocyclic carbenes (NHCs) are still developing as unique reactions that allow carbon–carbon and carbon–heteroatom bond formation. Sophisticated procedures lead to chemical compounds with complex molecular architecture. At the same time, NHC catalysis often enables highly stereoselective synthesis, which is important in the preparation of natural and/or biologically active compounds. Numerous review papers and books summarize the importance of NHC researchers’ achievements [1,2,3,4,5,6,7,8,9]. However, it is hard to look for any article mostly emphasizing the role of *N*-heterocyclic carbene catalysis in the history of organic chemistry. According to the authors, it is worth summarizing and emphasizing the impact of NHC catalysis on the development of name reactions, often developed in the nineteenth century.

This review contains a broad summary of name reactions enabled by NHCs. It is worth noting here that not all reactions are catalytic processes. Furthermore, this article compares historical reactions with contemporary organic chemistry, particularly emphasizing NHC catalysis. In some cases, it was also possible to highlight the applications of developed procedures or show unique processes based, e.g., on photoinduced reactions.

## 2. Appel Reaction

**Scheme 1:**
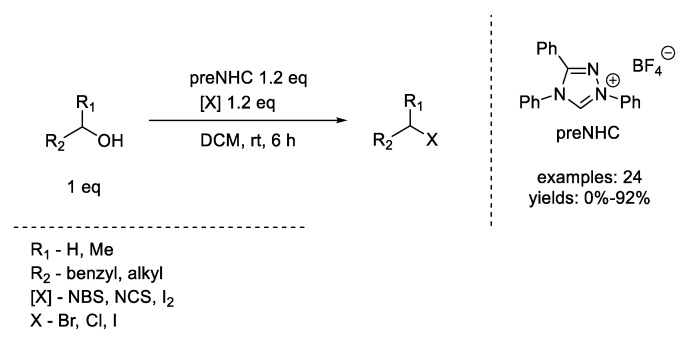
Reaction developed by Nguyen and Hussein.

In 2019, Nguyen reported an Appel-type reaction of alcohols with the use of NHCs instead of triphenylphosphine [10]. *N*-heterocyclic carbenes, very common organocatalysts, are used in this case as stoichiometric reagents (Scheme 1). Their application as phosphine mimetics is well-known in transition metal catalysis but it is important to remember the differences in electronic properties, generated steric hindrance, and complex stability between carbenes and phosphines [11,12]. Regardless of these differences, the authors proposed a mechanism fully identical to the mechanism developed by Appel in 1975 [13]. The reaction between the NHC and halogen source resulted in 5-halotriazolium halide being consumed afterward in the reaction with alcohol. The nucleophilic attack of halide anion on the generated 5-alkoxytriazolium intermediate led to the expected product and NHC-oxide.

## 3. Benzoin Condensation

Benzoin condensation is obviously not a name reaction, but it is associated with many names known to every organic chemist. Wöhler and Liebig reported the first benzoin reaction in 1832 [14], and a few years later, Zinin reduced the stoichiometric quantity of sodium cyanide to catalytic amounts [15,16]. Moreover, at the beginning of the 20th century, Lapworth supplemented the work of its antecedent with a reaction mechanism [17].

**Scheme 2:**
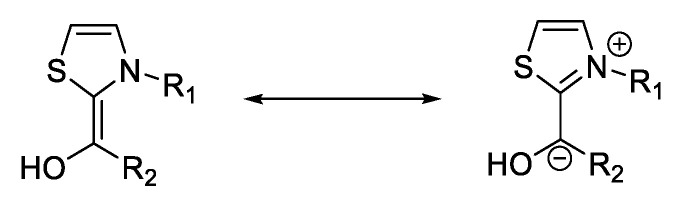
Breslow intermediate.

First, NHC-catalyzed benzoin condensation was reported by Ukai in 1943 [18]. It should be noted that this was historically the first *N*-heterocyclic carbene-catalyzed reaction. It gained special value when Breslow published its mechanism in 1958 [19]. One of the key intermediate products was named in honor of Ronald Breslow (Scheme 2). This means that benzoin condensation can be considered as a name reaction in the context of NHC catalysis.

The nature of the homo-benzoin reaction between benzaldehydes and the effect of substituents on the aromatic ring is well-known. Both electron-poor and electron-rich substrates are commonly used; however, strongly electron-withdrawing substituents can facilitate competitive redox processes [20,21,22]. Moreover, benzoin condensation is also possible between heteroaromatic aldehydes [23,24,25] as well as aliphatic aldehydes [23,26]. It should be noted that the benzoin reaction can be also carried out in a stereoselective manner. Highly enantioselective NHC-catalyzed benzoin condensation was firstly developed by Sheehan and Hunneman [27]. The thiazolylidene catalysts initially allowed a benzoin reaction with high enantioselectivity and low to moderate yields [28,29,30,31,32,33,34,35,36,37]. Exclusively, the design of catalysts based on triazolium salts allowed researchers to increase yields with the maintenance of high enantiomeric excesses [22,34,38,39,40,41,42,43,44,45]. 

**Scheme 3:**
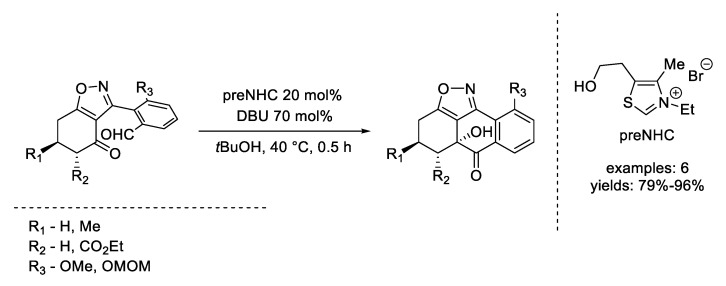
Reaction developed by Suzuki.

A greater challenge, however, is the cross-benzoin reaction. In the case of intramolecular cross-benzoin condensation, the reaction usually occurred between aldehyde and ketone. This approach enabled high chemoselectivity of the developed procedures. This was due to the fact that ketones do not react with *N*-heterocyclic carbenes to form Breslow intermediate. Such catalytic inertia of ketones arises, in part, from the keto-enol thermodynamics of Breslow intermediate. Instead, ketones are capable of reacting with Breslow intermediates generated from aldehydes. Firstly, the intramolecular cross-benzoin condensation in the synthesis of cyclic preanthraquinones was reported by Suzuki and co-workers in 2003 (Scheme 3) [46]. Later works significantly expanded the reaction scope and brought a great deal information about this condensation [47,48]. The reaction can occur between aliphatic or aromatic aldehydes and aliphatic or aromatic ketones. Nonetheless, α-methyl ketones take part in the reactions, leading to expected products with higher yields than more sterically hindered ketones. In this case, the homo-benzoin condensation is competitive. An intramolecular cross-benzoin reaction between the two aldehyde groups is also possible [49]. Unfortunately, chemoselectivity, in this case, is unsatisfactory. Even the macrocyclization reaction led to expected products with moderate yields [50]. Moreover, the reaction possibilities were expanded to stereoselective synthesis using triazolylidene catalysts [51,52,53,54,55,56]. It is worth noting that the enantiomeric excesses highly depended on the size of the newly generated ring. The best results were usually obtained for six-membered rings.

**Scheme 4:**
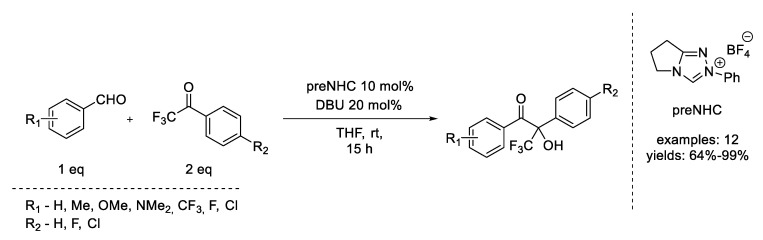
Reaction developed by Enders and Henseler.

In the case of intermolecular cross-benzoin condensation, the chemoselectivity also remained high when ketones were used as electrophiles. Firstly, such a reaction with the use of trifluoromethylketones was reported by Enders and Henseler in 2009 (Scheme 4) [57]. The authors obtained products with moderate to good yields and moderate enantiomeric excesses. The application of intermolecular cross-benzoin condensation was then expanded to α-ketoesters by Connon and Gravel [58,59]. Nonetheless, a much greater challenge is to obtain high chemoselectivity for the cross-benzoin reaction between aldehydes. Initially, the highest chemoselectivity was obtained for *ortho*-substituted aromatic aldehydes and α-substituted aliphatic aldehydes [60,61]. The same procedures were also carried out with sterically-unhindered aromatic aldehydes in later work [62]. Moreover, the use of triazolylidene as catalysts by Connon, Zeitler, and co-workers enabled the reduction of steric hindrance [63]. Furthermore, Gravel discovered that carrying out the reaction with piperidine-fused triazolylidene as catalyst allowed great chemoselectivity even in the case of condensation between simple benzaldehyde and acetic aldehyde [64,65]. It is also possible to control chemoselectivity using different catalysts under the same conditions. Yang and co-workers reported a reaction between aromatic aldehyde and acetaldehyde [66]. In the case of triazolium salt precatalyst, the Breslow intermediate was formed from acetaldehyde, whereas for thiazolylidene catalyst, the reaction of carbene with aromatic aldehyde was preferred. Moreover, the intermolecular cross-benzoin condensation enabled useful hydroxymethylation of aldehydes. Such a procedure of reaction between aldehydes and paraformaldehyde was reported by Glorius in 2011 [67]. It is worth noting that the chemoselective cross-benzoin reaction with the use of α-aminoaldehydes as electrophilic agents is also possible [68,69]. 

**Scheme 5:**
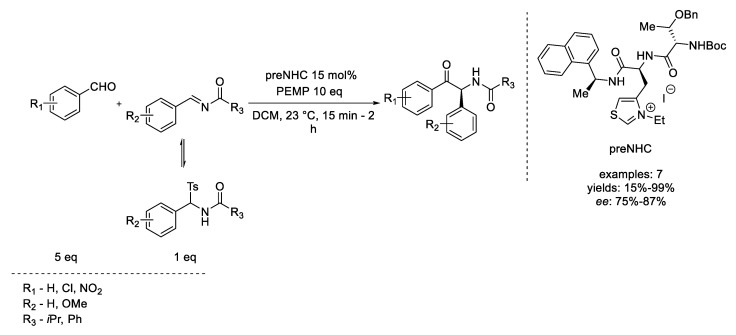
Reaction developed by Miller.

Another interesting aspect of the benzoin reaction is an *aza*-benzoin condensation. For the first time, such a reaction was reported by López-Calahorra in 1988 [70]. The condensation of iminium salts generated in the reaction of paraformaldehyde and morpholine or piperidine and aromatic aldehydes led to α-aminoketones with moderate yields. Later, the reaction scope was expanded to include acyl imines, Boc-protected imines, phosphinoylimines, *N*-arylimines, or *N*-aryliminium ions formed via a photoredox process [71,72,73,74,75]. Moreover, the first asymmetric *aza*-benzoin reaction catalyzed by thiazolylidene bonded to a peptide chain was reported by Miller and co-workers in 2005 (Scheme 5) [50]. Seven years later, Rovis et al. reported enantioselective condensation using triazolium salt as a precatalyst [76]. Furthermore, *aza*-benzoin condensation with ketimines as substrates is also possible [77,78,79]. Its additional advantage is the fact that possible racemization via enolization is blocked. However, it is worth noting that ketimines are less reactive species than aldimines in *aza*-benzoin condensation. 

## 4. Coates–Claisen and Ireland–Coates–Claisen Rearrangements

**Scheme 6:**
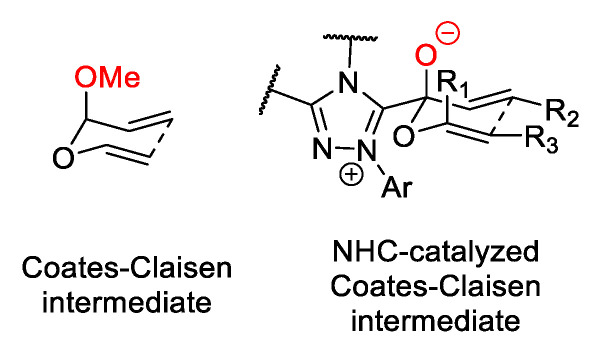
Similarity of C4 acceleration reported by Coates and Bode.

*N*-heterocyclic carbene-catalyzed Claisen rearrangements are also possible. However, the differences between the Claisen reaction reported firstly in 1912 [80,81,82] and the catalytic reactions referred to nowadays should be emphasized. Currently performed NHC-catalyzed Claisen rearrangements are based on the intermediate formed after enol addition to the acylazolium. The structure of this generated intermediate clearly indicates C4 acceleration reported by Coates (Scheme 6) [83]. Naturally, most 3,3-sigmatropic rearrangements catalyzed by NHCs are basically Coates–Claisen rearrangements. However, in brief, most of the similar reactions reported in currently published articles are called Claisen rearrangements. 

**Scheme 7:**
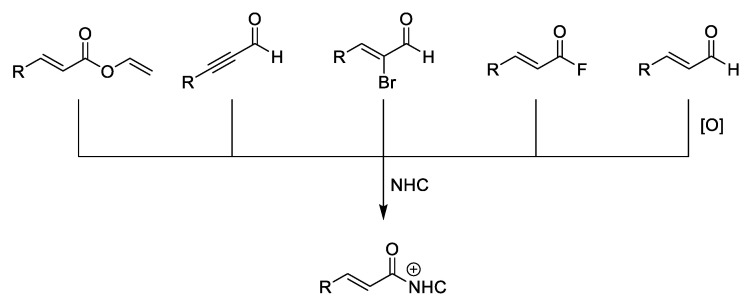
Formation of α,β-unsaturated acylazolium.

As mentioned, one of the mechanistic fundamentals of the Claisen-type reactions is the generation of α,β-unsaturated acylazolium (Scheme 7). Formation of this intermediate is usually carried out via the reaction of NHCs with α,β-unsaturated enol esters or ethers [84,85,86], ynals [87,88,89,90], 2-bromoenals [91,92,93,94], or acyl fluorides [95,96]. Moreover, formation α,β-unsaturated acylazolium is possible via two-electron oxidation of Breslow intermediate [97,98,99,100,101,102,103]. 

**Scheme 8:**
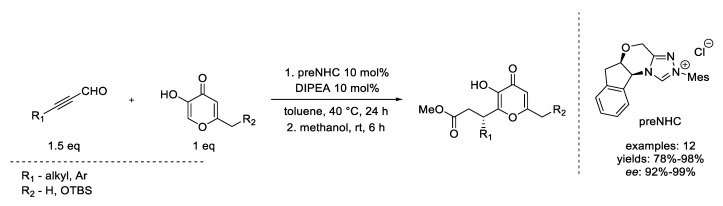
Reaction developed by Bode and co-workers.

The first NHC-catalyzed Coates–Claisen rearrangement was reported by Bode and co-workers in 2010 (Scheme 8) [89]. The reaction of ynals with kojic acids led to unstable dihydropyranones. The subsequent ring-opening through alcoholysis allowed the authors to obtain stable products without deterioration of enantiomeric excesses. The reaction began by the generation of α,β-unsaturated acylazolium, as noted above. Further reaction steps were acylation of enol, Claisen rearrangement of the resulting intermediate, tautomerization, and secondary lactonization. Moreover, the authors also used substrates other than kojic acids, but the results proved to be unsatisfactory. Two years later, Bode reported the broad extension of the substrate scope, including 2-naphthols [104]. In their case, the reaction stopped partially at the enol acylation step. Enantiomeric excess also remained moderate. The problem was solved in 2015 by You using the L-phenylalanine-derived catalyst [105].

Nevertheless, the Claisen-type reaction model had much wider application. Yu in 2012 showed the possibility of using diketones as sources of enol and α,β-dibromoaldehydes as precursors of α,β-unsaturated acylazoliums [91]. In addition, Bode significantly developed the subject of the possibilities of using acyclic enamines in the *aza*-Claisen rearrangement [106]. Rafiński and co-workers reported recently the first *aza*-Claisen reaction of cyclic enamines in the form of 6-amino-uracils [107]. Not much later, Biju showed interesting C2-functionalization of 3-aminobenzofurans via *aza*-Claisen rearrangement [108].

**Scheme 9:**
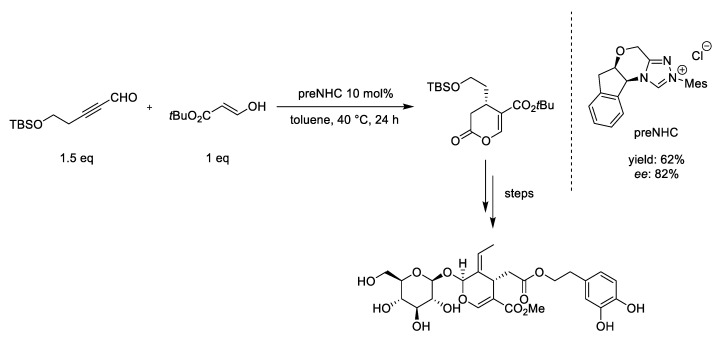
Reaction developed by Liu and co-workers.

Computational investigations [109], spectroscopic and kinetic studies of the mechanism [110], and a comprehensive discussion of the impact of the catalyst structure [111] have significantly increased knowledge of this process. Furthermore, an additional advantage is the example of use in total synthesis. Liu and co-workers used the Coates–Claisen reaction in the synthesis of a dihydropyran core for oleuropein based secoiridoids (Scheme 9) [112]. 

**Scheme 10:**
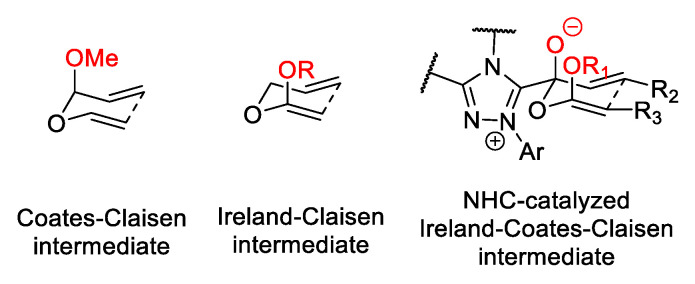
Similarity of C4 and/or C2 acceleration reported by Coates, Ireland, and Lupton.

*N*-heterocyclic carbene catalyzed Ireland–Coates–Claisen rearrangement is also possible. Such a reaction is slightly different from the Coates–Claisen variant reported by Bode [89]. The difference is a C2 activation similar to that reported by Ireland (Scheme 10) [113]. Moreover, acylazolium intermediate indicates also C4 acceleration reported by Coates [83].

**Scheme 11:**
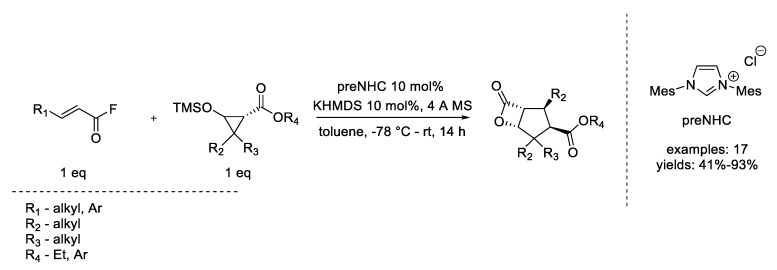
Reaction developed by Lupton and co-workers.

The first and only NHC-catalyzed Ireland–Coates–Claisen rearrangement was reported by Lupton in 2012 (Scheme 11) [114]. Acylazolium formation from acyl fluorides and releasing fluoride anion triggered desilylation of TMS-protected substrate. The subsequent retro-aldol reaction led to reactive enolate. Generation of hemiacetal and afterward Ireland–Coates–Claisen rearrangement is provided to the next intermediate, which undergoes aldol cyclization and lactonization to give a final product with good diastereoselectivity. Unfortunately, the authors decided to synthesize racemic mixtures due to competitive reactions which occur when the chiral sterically hindered catalyst is used.

## 5. *oxy*-Cope Rearrangement

The Cope rearrangement was discovered by Arthur C. Cope in 1940 [115]. This reaction in its nature is a thermal-induced isomerization of 1,5-dienes. However, in the *oxy*-Cope rearrangement [116], a substrate has a hydroxyl group on the sp^3^ carbon in position 3. This creates a product—enol or ketone, depending on keto-enol tautomerization. Isomerization is faster in this type of reaction, and it can take place at a lower temperature [117,118,119,120].

Moreover, the tautomerization allows the annulation processes to take place, so the *oxy*-Cope rearrangement is often one of the steps in multistep reactions. This is especially significant in obtaining complex compounds of biological importance. The mechanism is widely used in combination with benzoin condensation, called benzoin-*oxy*-Cope rearrangement, and with nitrogen instead of oxygen as *aza*-benzoin-*oxy*-Cope.

**Scheme 12:**
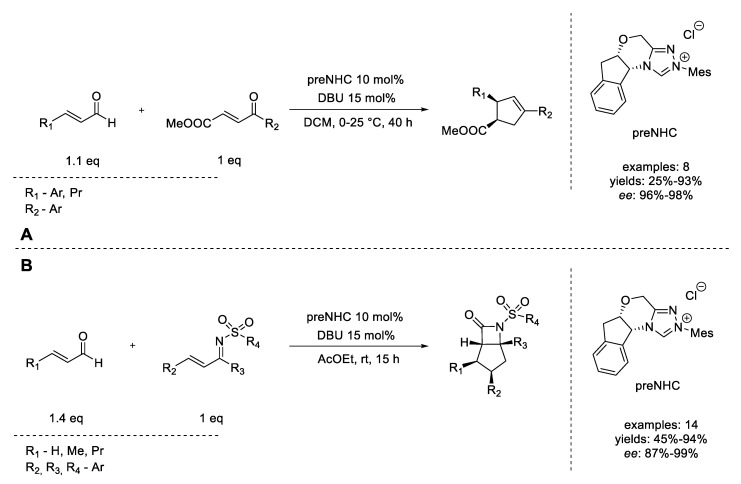
Reactions developed by Bode and co-workers. (**A**): 2007; (**B**): 2008.

Bode et al., based on their research, conducted a comparison [121] in which they found that some types of reaction prefer a specific *N*-heterocyclic carbene backbone as a catalyst. The *oxy*-Cope reaction is in general performed by employing triazolium salts as the NHC precursors. An example of this finding is cyclopentene forming annulation [122] or synthesis of bicyclo-β-lactams (Scheme 12) [123].

**Scheme 13:**
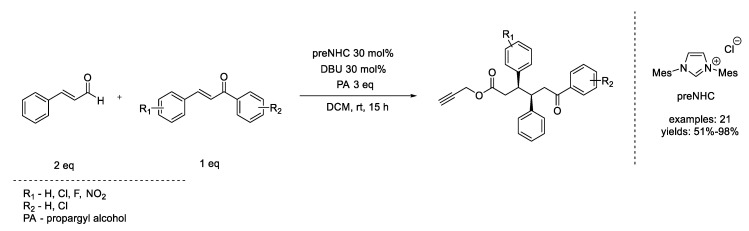
Reaction reported by Ma and co-workers.

Ma and co-workers implemented an interesting innovation in domino synthesis by using a three-component mixture catalyzed by NHC to obtain 1,6-dicarbonyl compound (Scheme 13) [124]. This cascade includes crossed-benzoin/*oxy*-Cope rearrangement/esterification processes. It is noteworthy that the catalyst precursor is no longer triazolium but imidazolium salt. Furthermore, ε-ketoesters are relevant in the total synthesis of natural compounds like prostaglandin A2 (PGA) [125], amphoteronolide B [126], or bilobalide [127,128]. 

## 6. Diels–Alder Reaction

**Scheme 14:**
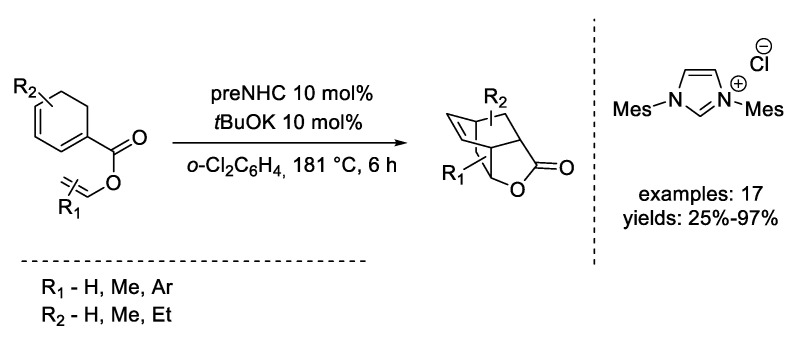
Reaction developed by Lupton and Kowalczyk.

A pure Diels–Alder reaction between conjugated diene and vinyl dienophile [129] was firstly reported by Lupton in 2014 (Scheme 14) [130]. Addition of an NHC catalyst to vinyl esters of cyclohexa-1,3-diene-1-carboxylic acid resulted in the creation of α,β,γ,δ-usaturated hemiacetal azolium intermediate. Further olefin isomerization of the resulting intermediate enabled an intramolecular Diels–Alder reaction. Moreover, the authors proved additional Lewis-base catalysis of the developed cascade olefin isomerization/Diels–Alder reaction. Furthermore, the possibility of derivatization of obtained tricyclic products rendered the developed procedure particularly worthwhile. 

**Scheme 15:**
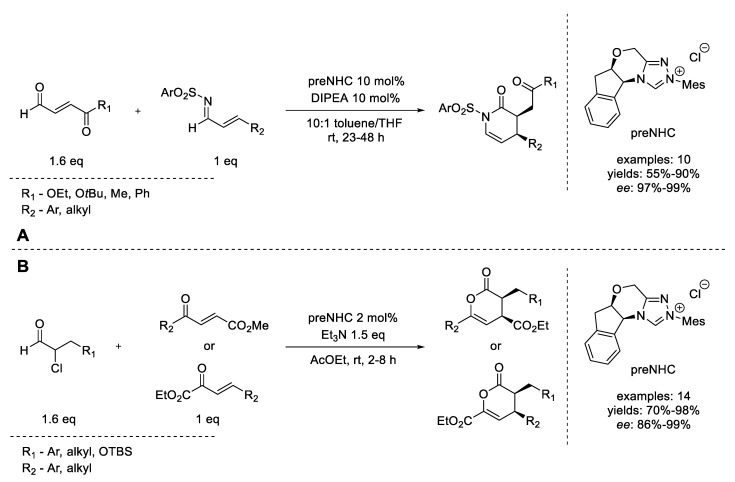
Reactions developed by Bode and co-workers. (**A**): June, 2006; (**B**): October, 2006.

A hetero-Diels–Alder reaction in the case of *N*-heterocyclic carbene catalysis is significantly more often encountered in the literature. Azadiene and oxodiene Diels–Alder reactions were firstly developed by Bode and co-workers in 2006 (Scheme 15A) [131,132]. In the case of azadiene-Diels–Alder reaction, β-EWG-substituted α,β-unsaturated aldehyde formed (*Z*)-enolate after the addition of the NHC catalyst. Such created dienophile reacts with α,β-unsaturated imine. The oxodiene-Diels–Alder reaction reported a few months later occurred in a similar way. In this case, α-chloroaldehyde and β-EWG-substituted α,β-unsaturated ketone or unsaturated α-ketoester were used as substrates (Scheme 15B). In both examples, the reactions occurred in a highly stereoselective manner, up to 99% *ee*.

**Scheme 16:**
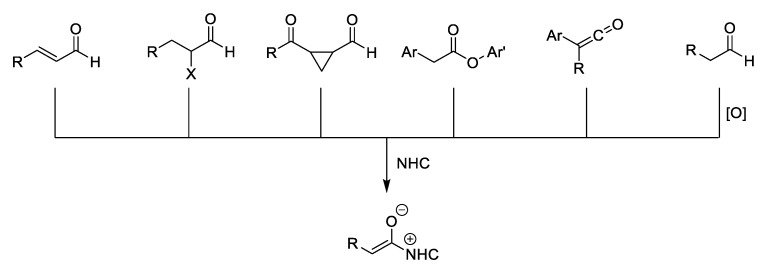
Formation of (*Z*)-enolate.

The enolate equivalent can arise not only from β-EWG-substituted α,β-unsaturated aldehyde or α-chloroaldehyde [133,134,135] but also from ketenes [136,137], cinnamaldehydes [138], functionalized formylcyclopropanes [139], esters [140], or simple saturated alkyl aldehydes under oxidizing conditions [141] (Scheme 16). 

**Scheme 17:**
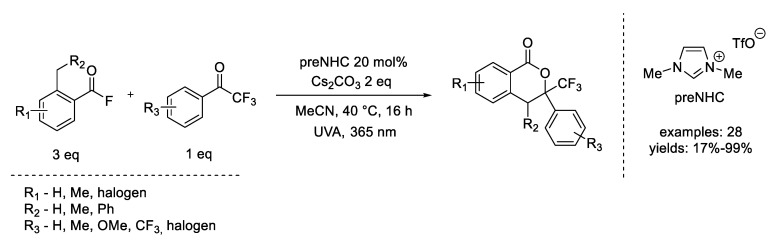
Reaction developed by Hopkinson and co-workers.

The work recently published by Hopkinson and co-workers merged light-mediated transformations with *N*-heterocyclic carbene catalysis [142]. Generated via the addition of NHC to benzoic acid fluoride, benzoyl azolium salt was changed into the biradical-like excited state during irradiation with UVA light. After rearrangement during relaxation, a diene-type intermediate was formed. Then, cycloaddition with 1,1,1-trifluoroacetophenones led to the hetero-Diels–Alder reaction product (Scheme 17). 

## 7. Michael Addition

A Michael reaction is an 1,4-addition type of conjugated addition. This reaction was discovered by Arthur Michael in 1887 [143]. It takes place between active methylene with α,β-unsaturated carbonyl compound, leading to C-C bond formation. Furthermore, asymmetric Michael addition has been known since the 1980s [144,145,146,147,148,149]. In addition, the umpolung effect forced by imines is known to take place in this type of synthesis [150].

Nevertheless, the crucial problem at those times, and also nowadays, was the low enantioselectivity and effectiveness of synthesis and the high cost. Consequently, when the first stable *N*-heterocyclic carbene (NHC) was discovered [151] and used in organocatalysis, new possibilities were introduced, not only in improving standard mechanisms but also by making slight changes to obtain a large effect during synthesis.

**Scheme 18:**
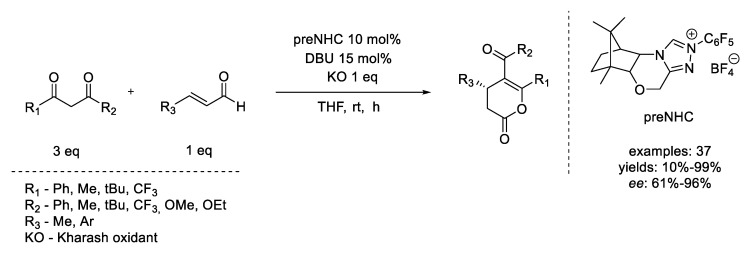
Reaction developed by You and co-workers.

Michael additions enabled by *N*-heterocyclic carbenes can be divided into a few types in terms of their pathways. The first type is the intermolecular reaction. It could be argued that the classic type of the mentioned addition of two molecules has been known for years. Despite this, with few exceptions [152,153], the standard Michael reaction is not the subject of modern research on *N*-heterocyclic carbene catalysis. There are many simple modifications of the process that lead to advanced mechanisms or subsequent reactions. For example, oxidizing the Breslow intermediate enables the annulation process [154]. (Scheme 18). It is worth noting that this reaction is confusingly similar to the Coates–Claisen rearrangement. However, the authors postulate that the reaction occurs via 1,4-Michael addition. Moreover, it gives useful intermediates for γ-lactones, benzenoids, or pyridine synthesis [155,156].

The chiral product also can be obtained with non-chiral 1,4-dimethyl triazolium carbene as a catalyst and chiral β-ketoamide as Michael donor. The reaction developed by De Sarkar and Struder takes place with DBU in THF under oxidative conditions [157].

**Scheme 19:**
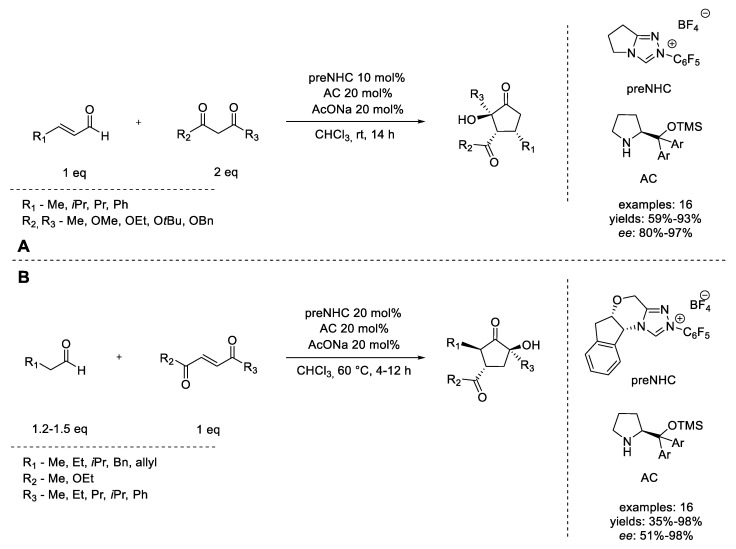
Reactions developed by Rovis and co-workers. (**A**): 2009; (**B**): 2011.

Another type of annulation by Michael reaction was demonstrated by Rovis and co-workers. Using a multi-catalyst mixture (proline derivative + NHC), they obtained [3+2] cyclic adduct [158,159] (Scheme 19). The first step is addition catalyzed by a proline derivative and the second is cyclization enabled by NHC. Similar research was performed by Ender’s group. The reaction takes place between enals and β-oxo sulfones, with yields and *ees* of up to 99% [160].

**Scheme 20:**
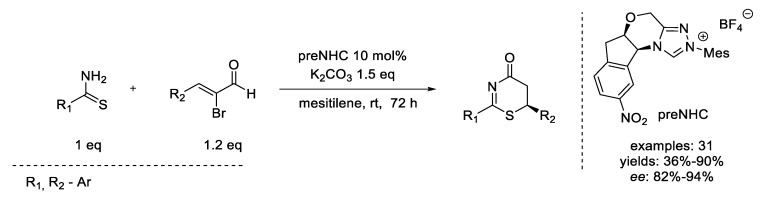
Reaction developed by Ghosh and co-workers.

Hetero-Michael reactions catalyzed by NHCs are also possible. A peculiar example of NHC catalysis also involving sulfur constituted the most recent research of Ghosh et al., in which *thia*-Michael addition is the first step of [3+3] annulation, starting from 2-bromoenals and thioamides [161] (Scheme 20). Nitrogen can also take part in a Michael reaction like *aza*-Michael addition in, for example, [3+4] annulation [94,162].

**Scheme 21:**
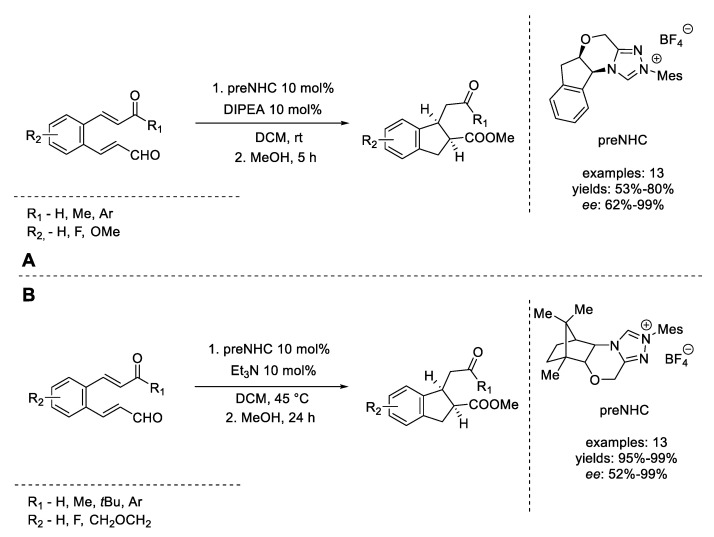
Reactions developed by Scheidt and co-workers (**A**) and You and co-workers (**B**).

A widely used example of 1,4-addition is the reaction that takes place in the same molecule giving the cyclic product. Often, an intramolecular Michael reaction is the first step in generating a more complex structure, as in, for example, the synthesis of 1,5-dicarbonyl compounds [163,164] (Scheme 21). Moreover, there is some research about the intramolecular synthesis of preanthraquinones from functionalized isoxazoles [46].

**Scheme 22:**
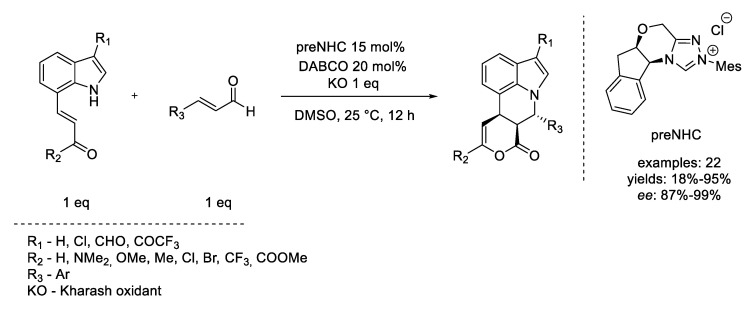
Reaction developed by Biju and co-workers.

Double Michael addition, also called a cascade Michael–Michael reaction, is a powerful method for the synthesis of complex natural compounds. Thus, synthesis of the pyrroloquinoline derivatives by *aza*-Michael–Michael cascade was the latest research conducted by Biju and co-workers [165] (Scheme 22).

Michael addition, as we can see above, is often combined with other types of reactions. The combination of Michael reaction and aldol condensation is called Robinson annulation [166]. Other types of Michael-type cascade reactions are, for example, benzoin-Michael–Michael cascade [167], Stetter–aldol–Michael cascade [168,169], and others [170,171,172,173].

## 8. Mitsunobu Reaction

**Scheme 23:**
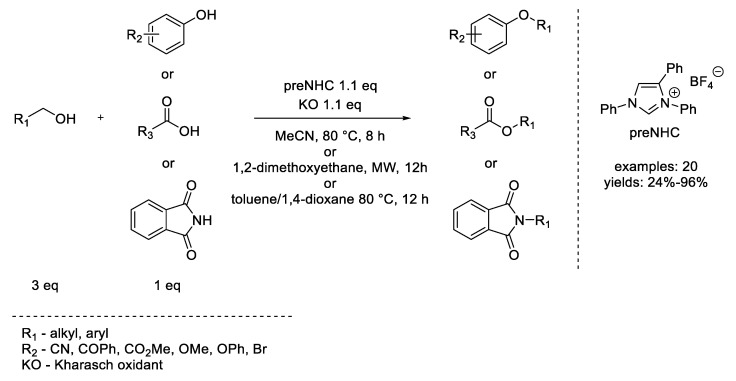
Reaction developed by Suzuki and co-workers.

A Mitsunobu reaction [174,175,176] with the use of *N*-heterocyclic carbenes as phosphine mimetics was reported a few years before a similar Appel reaction promoted by NHCs (Scheme 23) [177]. Suzuki and co-workers developed a protocol based on the reaction of alcohols with NHCs under oxidizing conditions, using the oxidant reported by Kharasch [178]. It should be emphasized that NHC is also used in this case as the stoichiometric reagent. The strong similarity to the Appel reaction lies in the formation of a 5-alkoxytriazolium intermediate and the subsequent nucleophilic attack on the resulting molecule. Expected aryl-alkyl ethers were obtained at elevated temperatures, with moderate to high yields. Several derivatives of alkylated phthalimide were obtained in a similar manner. Moreover, the developed protocol allowed the synthesis of esters under microwave (MW) irradiation, with moderate yields. 

## 9. Morita–Baylis–Hillman Reaction

**Scheme 24:**
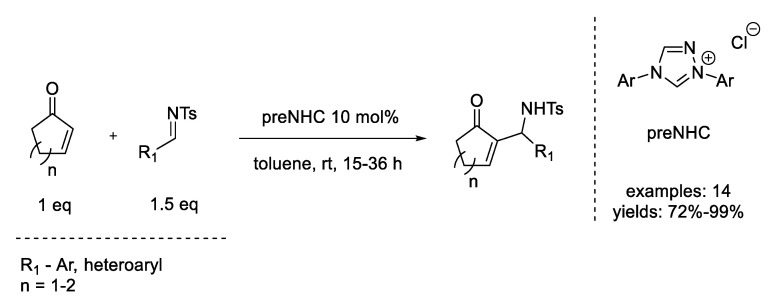
Reaction developed by Ye and co-workers.

*N*-heterocyclic carbenes proved to be efficient catalysts for the *aza*-Morita–Baylis–Hillman (*aza*-MBH) reaction [179,180]. This reaction involves the coupling of the activated alkene with an imine. In 2007, Ye and co-workers reported the reaction of cyclic enones with *N*-tosylarylimines (Scheme 24) [181], in which the free carbene added to the Michael acceptor formed an enolate. Then, enolate after reaction with imine, protonation, deprotonation, and release of NHC formed MBH adducts with high yields.

The enantioselective NHC-catalyzed *aza*-Morita–Baylis–Hillman reaction was presented in 2008 by Ye et al. [182]. Unfortunately, the reaction of cyclopent-2-enone with a *N*-tosylphenylmethanimine yielded a product with low enantioselectivity (up to 44% *ee*). 

**Scheme 25:**
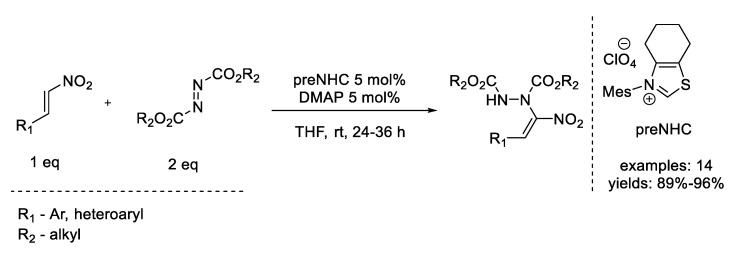
Reaction developed by Ye and co-workers.

The novel *N*-heterocyclic carbene-catalyzed Morita–Baylis–Hillman (MBH) reaction of β-substituted nitroalkenes and azodicarboxylates was reported in 2013 by Ye et al. (Scheme 25) [183]. Earlier, Namboorhiri et al. reported MBH reaction of β-aryl nitroethylenes and activated alkenes catalyzed by imidazole, but they used up to 100 mol% of catalyst [184]. In the case of NHC catalysis reported by Ye and co-workers, a catalyst reduction of up to 5 mol% allowed the authors to obtain final products with excellent yields [183].

## 10. Rauhut–Currier Reaction

**Scheme 26:**
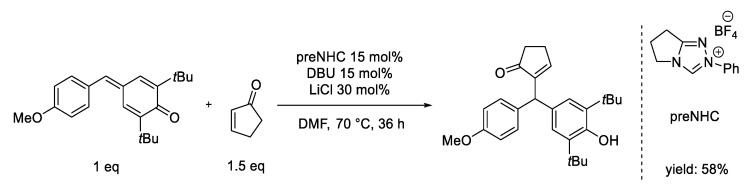
Reaction developed by Anand and co-workers.

The first report of an NHC-catalyzed Rauhut–Currier (RC) reaction [185] which involves the coupling of an active alkene (latent enolate) to a Michael acceptor describes the cooperative use of NHCs as catalyst or initiator [186,187]. The direct application of NHC as the sole catalyst was presented by Anand in 2018 (Scheme 26), but this reaction was not efficient without the use of LiCl, which stabilizes the produced enolate [188]. 

**Scheme 27:**
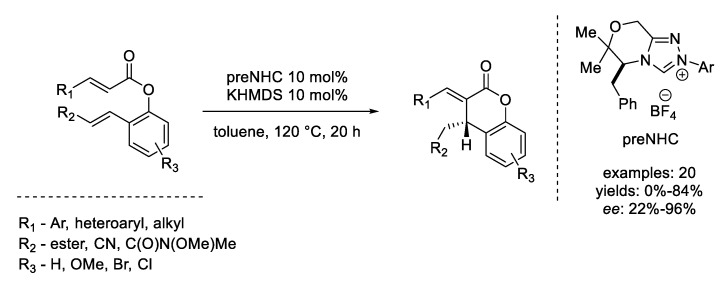
Reaction developed by Lupton and co-workers.

In 2019, Lupton and co-workers exploited the high nucleophilicity of *N*-heterocyclic carbenes to achieve intramolecular RC reaction of bis(enoate) substrates (Scheme 27) [189]. The addition of NHC resulted in the formation of an enolate, which then cyclized to form the lactone, with a new C–C bond between the α-position of one activated alkene and the β-position of the second alkene. 

## 11. Staudinger Cycloaddition

Since the first report on the Staudinger ketene-imine cycloaddition in 1907 [190], many effective ways to modify the reaction conditions have been demonstrated. NHC catalysis proved to be an effective way to change reaction conditions. 

**Scheme 28:**
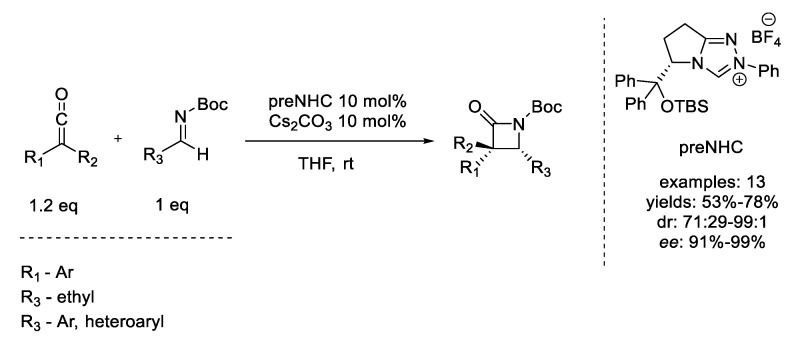
Reaction developed by Ye and co-workers.

The first NHC-catalyzed Staudinger cycloaddition was presented in 2008 by Ye and co-workers [191]. Authors described the reaction between ketenes and *N*-protected aldimines (Scheme 28). In this case, imines are strongly electrophilic; therefore, initially, carbene reacts with ketene to generate a zwitterionic azolium enolate. It reacts further with the imine and forms the final product after cyclization [192]. *Cis*-β-lactams have been obtained with good yields, good diastereoselectivity, and excellent enantioselectivity [191]. 

The zwitterionic azolium enolate may also react with a carbonyl group to produce a β-lactone. Recently, such a version of the Staudinger reaction using trifluoromethyl ketones [193], 2-oxoaldehydes [194], aldehydes [195], or isatins [196] has been presented. Other approaches include the reaction of ketenes with azodicarboxylates [197], nitroso compounds [198], or *N*-sulfinylanilines [199]. 

**Scheme 29:**
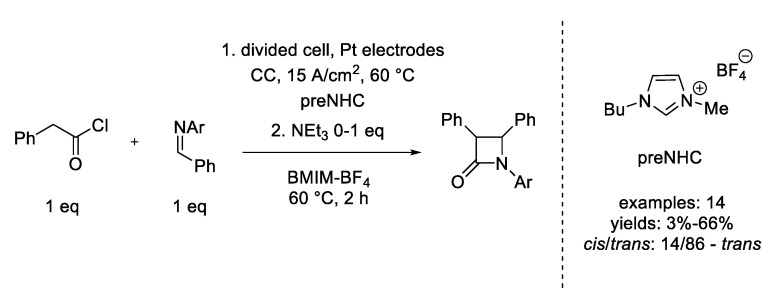
Reaction developed by Feroci and co-workers.

In 2010, Feroci et al. applied an innovative method of conducting Staudinger cycloaddition reaction between ketene, generated by dehydrohalogenation of an acyl halide and a non-electrophilic *N*-aryl aldimine in 1-butyl-3-methylimidazolium tetrafluoroborate (BMIM-BF_4_) (Scheme 29) [200]. The ionic liquid played the dual role of solvent and precatalyst for electrochemical carbene generating. The authors are currently studying the role of this electrogenerated carbene in the reaction mechanism [201,202]. Final products have been obtained predominantly as *trans*-β-lactams with a good diastereomeric ratio. 

## 12. Stetter Reaction 

**Scheme 30:**
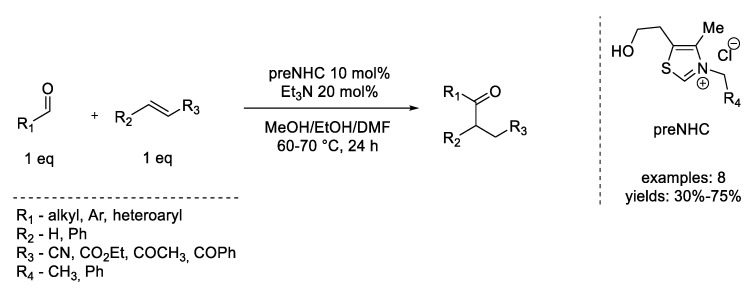
Reaction developed by Stetter and Kuhlmann.

In 1974, Stetter and Kuhlmann noticed that the thiazolium salt in the presence of a base catalyzes the reaction of α,β-unsaturated ketones, esters, and nitriles with aliphatic, aromatic, and heterocyclic aldehydes (Scheme 30) [203]. Such a reaction, later called the Stetter reaction, is a good synthetic tool for the construction of 1,4-bifunctional compounds. Furthermore, the Stetter reaction has been successfully used as a model reaction in the developing of NHC catalysts [204,205,206,207,208]. 

The acyl anion equivalent (Breslow intermediate) generated from aldehyde after the addition of NHC can react with various α,β-unsaturated compounds, called Michael acceptors, to form 1,4-dicarbonyl compounds or other derivatives such as ketophosphonates [209], nitroketones [210], or ketonitriles [211]. Other approaches involved the generation of *aza*-Breslow intermediate via imine umpolung (*aza*-Stetter) [212] or Breslow intermediate from acylsilanes (*sila*-Stetter) [213].

**Scheme 31:**
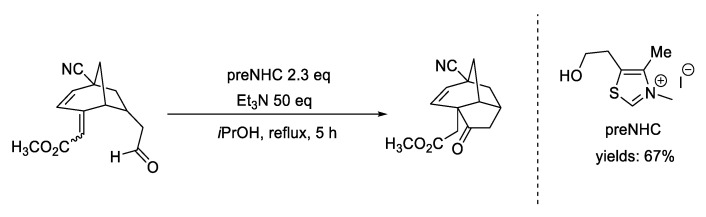
Reaction developed by Trost and co-workers.

The intramolecular Stetter reaction was presented firstly in 1979 by Trost et al. (Scheme 31) [214]. In total, 2.3 equivalents of the thiazolium salt were used in this reaction to obtain a product with a good yield. Additionally, this work is an example of the first formation of a quaternary stereogenic center via the Stetter reaction.

**Scheme 32:**
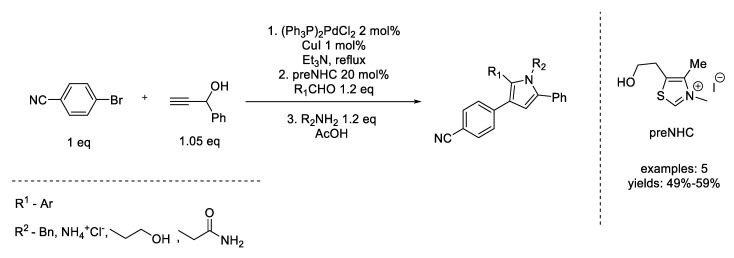
Reaction developed by Müller and co-workers.

The Stetter reaction can be combined with Paal–Knorr synthesis of furans and pyrroles [215,216], in which 1,4-difunctionalized Stetter product undergoes condensation. In 2001, Müller and coworkers reported the synthesis of pyrroles via a one-pot, three-step, four-component process by a coupling-isomerization Stetter–Paal–Knorr pathway (Scheme 32) [217]. 

Another example of the synthesis of pyrroles utilizing a Stetter–Paal–Knorr strategy was presented by Scheidt [218]. The *sila*-Stetter reaction of acylsilanes with unsaturated ketones generated 1,4-dicarbonyl compounds in situ. Then, the Paal–Knorr reaction with various amines afforded desired pyrroles with good yields. 

**Scheme 33:**
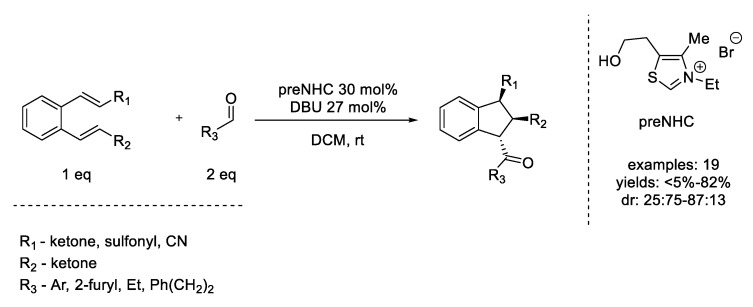
Reaction developed by Gravel and co-workers.

A cascade reaction involving NHC-catalyzed Stetter reaction was presented in 2009 by Gravel and co-workers (Scheme 33) [219]. An enolate intermediate, generated from Breslow intermediate and Michael acceptor, may undergo two possible pathways to form indane by nucleophilic attack onto an appropriate electrophile and cyclization or by forming the simple Stetter product, which could afford final product in basic conditions. The indane derivatives were obtained in good yield and good diastereomeric ratio. 

**Scheme 34:**
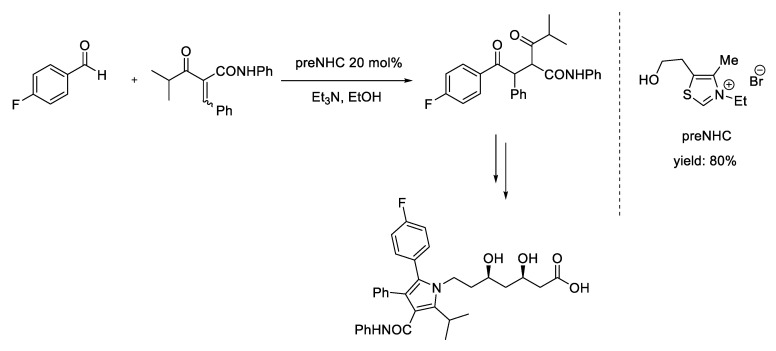
Reaction developed by Roth and co-workers.

The Stetter reaction is a useful and effective tool used as one of the steps in the synthesis of complex organic molecules such as natural products, e.g., dihydrojasmone and *cis*-jasmone [220], *trans*-sabiene hydrate [221], and (+)-monomorine I [222], and medicinally relevant non-natural products. The 1,4-diketone intermediate required for the synthesis of the compound sold under the trade name Lipitor was synthesis by the Stetter reaction (Scheme 34) [223]. This transformation was used in the industrial route [224].

## 13. Wallach Reaction

Described by Otto Wallach in 1873 [225], oxidation of chloroaldehydes to carboxylic acids was the first step of later redox-type organocatalytic reactions of α-reducible aldehydes [226,227,228]. Nowadays there are many variations of this reaction involving not only aldehydes but also alcohols and carboxylic acids as substrates.

**Scheme 35:**
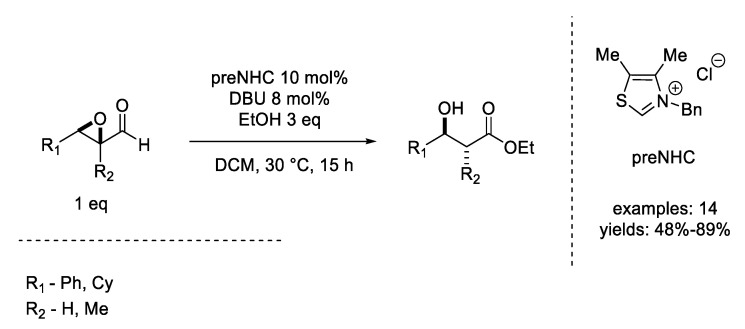
Reaction developed by Bode and co-workers.

The first thiazolium-catalyzed Wallach-type reaction was announced by Castells’s group [229] almost 100 years after Wallach’s discovery. They obtained methyl esters from corresponding aryl aldehydes. Meanwhile, the year 2004 represented a breakthrough in *N*-heterocyclic carbene catalysis because of many independent reports of fundamental reaction types. Bode and co-workers were among these pioneering researchers [230] reporting an NHC-catalyzed Wallach-type reaction of epoxyaldehydes (Scheme 35).

**Scheme 36:**
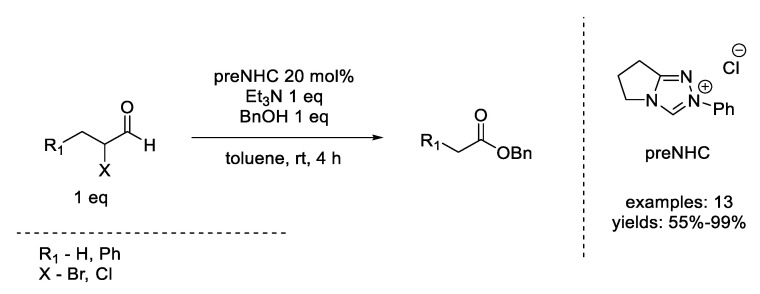
Reaction developed by Rovis and co-workers.

Only a few issues later [231] in the same journal, research by Rovis and co-workers was published. They obtained esters from α-halogenaldehydes in the presence of triazolium salt and triethylamine in toluene (Scheme 36).

Further research led to obtaining α-chloroesters [232] by the same method and also other types of economical redox esterification of enals like α-hydroxy or α-amino esters [233,234,235]. However, to prevent C–C bond formation from Breslow intermediate instead of its oxidation, the proper base should be used [236]. 

**Scheme 37:**
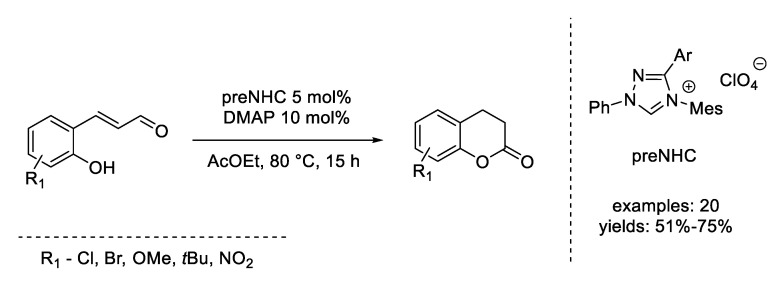
Reaction developed by Zeitler and Rose.

*N*-heterocyclic carbenes are widely used in many annulation processes; thus, it was obvious that the Wallach-type mechanism could also be applied in this type of synthesis. According to this method, Zeitler and Rose in 2009 obtained 3,4–dihydrocumarins [237] (Scheme 37).

## 14. Summary

*N*-heterocyclic carbenes enable numerous name reactions, although sometimes, at first glance, it is difficult to find historical mechanisms there. Nonetheless, we managed to highlight these similarities and show NHC catalysis as a development of the original name reactions. This is especially helpful for readers learning about *N*-heterocyclic carbene-based organocatalysis and provides knowledge of the basics of organic chemistry. We hope that this presentation of the evolution of organic chemistry will be an inspiration for the further development of organocatalytic applications of *N*-heterocyclic carbenes.

## Acronyms

CC	constant current
DBU	1,8-diazabicycloundec-7-ene
DCM	dichloromethane
DIPEA	diisopropylamine
DMSO	dimethylsulfoxide
KHMDS	potassium hexamethyldisilazide
KO	Kharasch oxidant (3,3’,5,5’-tetra-*tert*-butyldiphenoquinone)
NHC	*N*-heterocyclic carbene
PEMP	1,2,2,6,6-pentamethylpiperidine
preNHC	NHC precatalyst
THFTMS	TetrahydrofuranTrimethylsilyl group
